# A prospective cohort study of dietary patterns of non-western migrants in the Netherlands in relation to risk factors for cardiovascular diseases: HELIUS-Dietary Patterns

**DOI:** 10.1186/1471-2458-11-441

**Published:** 2011-06-07

**Authors:** Louise H Dekker, Marieke B Snijder, Marja H Beukers, Jeanne HM de Vries, Henny AM Brants, Evelien J de Boer, Rob M van Dam, Karien Stronks, Mary Nicolaou

**Affiliations:** 1 Department of Public Health, Academic Medical Centre, University of Amsterdam, PO Box 22660, 1105 AZ, Amsterdam, the Netherlands; 2 National Institute for Public Health and the Environment (RIVM), PO Box 1, 3720 BA Bilthoven, the Netherlands; 3 Division of Human Nutrition, Wageningen University, PO Box 8129, 6700 EV Wageningen, the Netherlands; 4 Departments of Epidemiology and Public Health and Medicine, Yong Loo Lin School of Medicine, National University of Singapore, 16 Medical Drive, Singapore 117597, Singapore

**Keywords:** Food frequency questionnaire, dietary patterns, ethnicity, cardiovascular diseases

## Abstract

**Background:**

In Western countries the prevalence of cardiovascular disease (CVD) is often higher in non-Western migrants as compared to the host population. Diet is an important modifiable determinant of CVD. Increasingly, dietary patterns rather than single nutrients are the focus of research in an attempt to account for the complexity of nutrient interactions in foods. Research on dietary patterns in non-Western migrants is limited and may be hampered by a lack of validated instruments that can be used to assess the habitual diet of non-western migrants in large scale epidemiological studies. The ultimate aims of this study are to (1) understand whether differences in dietary patterns explain differences in CVD risk between ethnic groups, by developing and validating ethnic-specific Food Frequency Questionnaires (FFQs), and (2) to investigate the determinants of these dietary patterns. This paper outlines the design and methods used in the HELIUS-Dietary Patterns study and describes a systematic approach to overcome difficulties in the assessment and analysis of dietary intake data in ethnically diverse populations.

**Methods/Design:**

The HELIUS-Dietary Patterns study is embedded in the HELIUS study, a Dutch multi-ethnic cohort study. After developing ethnic-specific FFQs, we will gather data on the habitual intake of 5000 participants (18-70 years old) of ethnic Dutch, Surinamese of African and of South Asian origin, Turkish or Moroccan origin. Dietary patterns will be derived using factor analysis, but we will also evaluate diet quality using hypothesis-driven approaches. The relation between dietary patterns and CVD risk factors will be analysed using multiple linear regression analysis. Potential underlying determinants of dietary patterns like migration history, acculturation, socio-economic factors and lifestyle, will be considered.

**Discussion:**

This study will allow us to investigate the contribution of the dietary patterns on CVD risk factors in a multi-ethnic population. Inclusion of five ethnic groups residing in one setting makes this study highly innovative as confounding by local environment characteristics is limited. Heterogeneity in the study population will provide variance in dietary patterns which is a great advantage when studying the link between diet and disease.

## Background

Evidence points out that non-Western migrants (hereafter referred as migrants) are likely to acquire the chronic disease patterns of the country to which they migrate [1]. In some cases migrants and their offspring have even higher rates of mortality and morbidity linked to cardiovascular diseases (CVD) compared to the host population [2-5]. Compared to the ethnic Dutch population, the prevalence of overweight and obesity has found the be higher among Surinamese, Turkish and Moroccan migrants [6], hypertension is higher in Surinamese of African origin [7], and type 2 diabetes is more common in Surinamese South Asian, Moroccan and Turkish migrants [8,9]. Differences in the diet between host populations and migrants may partly underlie the observed higher risk for CVD.

The study of diet-disease relationships is complicated by the fact that nutrients do not exist in isolation; they are obtained from foods, containing a mix of different nutrients and bioactive substances. Therefore, in addition to studying the role of individual nutrients, dietary patterns have become a focus for nutritional research [10,11]. It is expected that insight into the association between habitual dietary patterns and CVD risk may help in prioritizing public health efforts and provide a concrete basis for recommendations to improve food intakes. However, the increasing ethnically diverse nature of many populations in Europe complicates the assessment and analysis of representative dietary intake data for several reasons. First, population based studies measuring habitual dietary intake have often excluded migrants because, alongside communication issues, most research tools that are currently available have been developed for the host population and are not necessarily valid and critically assessed for their suitability in migrant groups [12]. Second, the accuracy of dietary intake data is limited, due to lack of food composition data on ethnic foods consumed by migrants in Europe [5].

In the Netherlands, dietary intake among some migrant groups has been assessed on a single occasion using 24 h recalls [13-17], in small study samples, and the comparison with the ethnic Dutch population has often been indirect [15,16]. Ethnic-specific tools to measure the habitual dietary intake of individuals from the predominant migrant groups are currently not available. As a result, we do not have insight into migrants' habitual dietary intakes, a necessary prerequisite for the assessment of dietary patterns and, ultimately, for making a valid link with risk factors for CVD. The aims of the present study are to therefore (1) to develop ethnic-specific food frequency questionnaires (FFQs) to measure habitual dietary intake of Surinamese of African, Surinamese of South Asian, Turkish, and Moroccan origin residents in Amsterdam, the Netherlands; (2) to assess the suitability and validity of these ethnic-specific FFQs; (3) to describe the dietary patterns of these migrant groups and of the ethnic Dutch population; (4) to explore the association between dietary patterns and risk factors for CVD; (5) to evaluate determinants of dietary patterns by investigating whether the dietary patterns differ within ethnic groups according to characteristics such as sex, social-economic status (SES), age (generation level), place of birth, acculturation and lifestyle.

This paper outlines the design and methods used in the HELIUS-Dietary Patterns study and describes a systematic approach to overcome the difficulties in the assessment and analysis of dietary intake data in ethnically diverse populations.

## Methods/Design

### Cohort

The HELIUS study (acronym for HEalthy LIfe in an Urban Setting), is a large prospective longitudinal cohort study in Amsterdam, the Netherlands, established with the primary aim to unravel the causes of (the unequal burden of) diseases across ethnic groups. HELIUS commenced in January 2011 and has an emphasis on CVD, mental health and infectious diseases; all major causes of the global burden of disease. The cohort will consist of 60,000 subjects originating from 6 different ethnicities: African origin Surinamese, South Asian origin Surinamese, Turkish, Moroccan, Ghanaian, and ethnic Dutch. The base population will consist of participants aged 18-70 years and will be randomly sampled according to ethnic origin through the municipality register of Amsterdam. Of each participant, a maximum of three family members (parents, siblings, children and partner) will be invited, until 10,000 subjects per ethnic group are included. Persons who are unable to give informed consent or persons without a general practitioner will be excluded.

In the HELIUS-Dietary Patterns study we will include a sub-sample of the HELIUS population. Specifically, 1000 individuals per ethnic group including African origin Surinamese, South Asian Surinamese, Turkish, Moroccan, and ethnic Dutch will be included. This will result in 5000 participants. HELIUS participants of Ghanaian origin will be excluded as there is no initial dietary intake data available for the development of a Ghanaian-specific FFQ. Cardiovascular risk factors (including among others, blood pressure and lipid levels) will be the main outcomes being tested with dietary pattern scores as the dependent variable. Due to the explorative nature of this study, our assumption is that we will have a multiple linear regression model which already includes 2 covariates. With a sample size of 1000 and alpha = 0.05, we will have an 80% power to detect an increase in R-squared of 0.016 due to the inclusion of dietary pattern scores in the model. Additionally, studies in which models are validated on independent datasets have shown that in many situations a fitted regression model is likely to be reliable when the number of predictors is less than the total sample size/20. Therefore we expect that a sample size of 1000 individuals per ethnic group will be sufficient to serve the aims of this study [18]. The HELIUS study and the present sub-study were approved by the AMC Medical Ethics Committee.

### Recruitment, inclusion and exclusion criteria

Participants of the HELIUS study that consent to taking part in additional evaluations will be eligible for the HELIUS-Dietary Patterns study. We will include participants from the HELIUS base population, i.e. family members will be excluded in order to avoid clustering. In addition we aim to include equal representation of men and women, of all age groups. Among the migrant groups we will also aim for equal representation of generations, i.e. generation level as defined by country of birth. Those born outside the Netherlands are first generation immigrants, those born in the Netherlands and having one or both parents born abroad are second generation immigrants and so forth [19]. Participants will be recruited by HELIUS personnel upon completion of the HELIUS physical examination.

### Study design

HELIUS-Dietary Patterns comprises of five principal phases, each with stand-alone aims. We will describe these phases one by one.

### Phase 1. Development of ethnic-specific Food Frequency Questionnaires

Experience with research among different ethnic groups leads us to choose the FFQ to assess habitual dietary intake. A low literacy level among our study population is likely to hamper the keeping of food diaries. Language barriers mean that bi-lingual interviewers will be necessary in this study; however, recruitment of highly trained bi-lingual interviewers to conduct either diet history or 24 hr recall interviews is difficult [6]. In contrast, FFQs are an acceptable tool for measuring habitual dietary intake in large scale epidemiological studies such as HELIUS-Dietary Patterns [20]. Importantly, FFQs can be administered by minimally trained bi-lingual interviewers, the interview time is relatively short and a single interview can provide data on usual food intakes.

Given the lack of available questionnaires for the measurement of the habitual food intake among migrants in the Netherlands, a major aim of HELIUS-Dietary Patterns is to develop three ethnic-specific FFQs to reflect the habitual diet of Turkish, Moroccan and Surinamese participants. Although the Surinamese group in HELIUS consists of two distinct ethnic groups (African origin and South Asian origin), similarities in the foods commonly eaten allow the design of a single questionnaire. An validated FFQ that has been recently developed for the ethnic Dutch population by the department of Human Nutrition at Wageningen University [21] will be used as a template. This FFQ will be adjusted for Turkish, Moroccan and Surinamese origin participants in order to reflect the foods they usually consume. In order to enhance comparability, the newly developed FFQs and the validated Dutch FFQ will undergo a final adjustment to optimize their comparability to each other. This process will be described in detail elsewhere (article in preparation). In short, the food items in the Dutch template FFQ will be adjusted on the basis of dietary intake data previously gathered by 24 hr recall interviews among 154 Moroccan (18-58 years old), 109 Surinamese (35-60 years old) and 175 Turkish (18-56 years old) migrants in the Netherlands [15,16,22]. This process will employ the methodology of the computerized Dutch FFQ-tool, a system to generate, apply and process FFQs [23]. First, food items will be selected on the basis of explained variance in nutrient intake without taking covariance from other food items into account. Second, we will select food items which highly contribute to the level of nutrient intake of the total population. Regarding the latter, the minimum set of food items that yield at least 80% of the intake is selected. Food items selected for inclusion in the FFQ are based on calculations performed for 20 nutrients (including all macronutrients, dietary fibre, vitamin A, B2, B12, C and D, folate, calcium and iron) in order to allow research into the link between CVD risk factors and both individual nutrients and dietary patterns.

### Phase 2. Suitability and validity of the ethnic-specific FFQs

Given the complexities of dietary intake, all dietary intake assessment methods are associated with measurement errors which affect estimated dietary intake and may obscure diet-disease risk associations. For this reason, and because of the relatively small sample size of the studies used to provide initial dietary intake data for the ethnic-specific FFQ development, additional steps are needed to ensure suitability, completeness and validity of the newly developed ethnic-specific FFQs.

### Phase 2a. Assessment of suitability of the ethnic-specific FFQs

Evaluation of face-validity, cognitive interviews and focus group discussions are approaches which can be applied to improve completeness and clarity of the FFQ [24-27]. These approached will all be applied in phase 2a.

Face-validity concerns whether the questionnaire looks like it is actually estimating what it is supposed to measure [28], and is usually assessed by experts on the investigated topic. In our case, the aim of the face-validity checking is to confirm with dieticians or nutritionists with a Turkish, Moroccan or Surinamese background whether (all) important ethnic specific food items are included in the FFQ.

Cognitive interviews involve the generation of verbal information about comprehension and difficulty while a subject is answering questions of the FFQ. This verbal information is subsequently used to improve the quality of questions [29]. The aim of the cognitive interviews (one-on-one think aloud interviews) is to identify cognitive problems when filling out the questionnaire among the population of interest and to enhance the clarity of the FFQs.

Finally, all specific components of the ethnic-specific FFQs will be evaluated among the target populations in focus group discussions as a triangulation and supplementation of the information gathered by face-validity checking and the cognitive interviews.

### Phase 2b. Assessment of validity of the ethnic-specific FFQs

With the absence of a golden standard, the use of nutrition biomarkers in dietary validation studies is attractive, because errors for biochemical markers and reporting methods can generally be assumed to be independent. Recovery biomarkers can be used to calculate absolute intakes over a specific time period, and as reference instruments in calibration studies. However, the limitation is that the currently available recovery biomarkers reflect only a very small proportion of the variation in dietary intakes that is of interest. Therefore, concentration biomarkers are often used to assess the validity of dietary intake instruments. Fatty acids and carotenoids have been found to play an important role in the link between diet and CVD and are therefore suitable considering our wish to explore this association in different ethnic groups [20,30,31]. In this validation study, we will estimate the associations between concentration biomarkers for (1) fatty acids profiles to evaluate the ability of the FFQ to measure fat intake, and (2) carotenoid profiles to validate fruit and vegetable intake.

We will perform this validation within a sub-sample of each ethnic group (total n≈750). Fasting blood samples will be drawn to measure biomarkers for the validation of the ethnic-specific FFQs. Specifically, in these blood samples we will indentify fatty acids with a focus on those that are largely of exogenous origin (e.g. n-3 and n-6 polyunsaturated fatty acids, *trans *fatty acids, and odd-numbered fatty acids) with meaningful concentrations (mean concentration >0.10%), accounting for >95% of total identified fatty acids. In addition, we will measure major carotenoids found in human plasma; β-carotene, α-carotene, lutein, lycopene, zeaxanthin and β-cryptoxanthin. There are hundreds of other carotenoids, but these are less commonly found in foods. Blood samples will be stored, which allows for future testing of other micronutrients, like folate.

When using a biochemical indicator as a reference, care must be taken to use an indicator that measures nutrient intake during the period evaluated by the diet assessment instrument being tested. The FFQs will assess dietary intake exposure of the last 4 weeks. The optimal specimen type for the measurement of biomarkers differs by dietary components of interest. For example for fatty acids, adipose tissue better reflects long-term intake than plasma, but for carotenoids, plasma biomarkers appear to reflect long-term intake at least as accurately as adipose tissue levels [32]. However, adipose tissue is rarely used in large epidemiologic studies because of the difficulty in obtaining the samples [33,34]. In this study, plasma will be used to collect biomarkers for total fat and fruit and vegetable intake. Validation studies indicate that plasma captures long-term intake reasonably well for fat, albeit lower than for adipose tissue [30]. Because homeostatic and metabolic processes influence biochemical indicator levels, the correlation between an indicator and an assessment of dietary intake can be taken as an estimate of the lower limit of the validity of the diet assessment method [35]. Validation of the FFQs using an additional method, preferably repeated 24 hour dietary recalls, would be ideal but falls outside the scope of the current project.

### Phase 3. Dietary pattern analysis

Dietary patterns will be examined for three reasons: (1) to characterize dietary behaviour; (2) to assess the nutritional quality in these dietary phenotypes in comparison with dietary recommendations for health and prevention of CVD); (3) to examine the relation between dietary patterns and risk factors for CVD (phase 4). The first reason has a descriptive purpose (e.g. who is eating the westernized diet and who is eating the traditional diet?). The second and the third involve hypothesis testing (e.g. is adherence to a particular diet associated with more favourable cardiovascular risk factors?).

Initially we have chosen to use a data-driven approach in this phase of HELIUS-Dietary Patterns. This approach is appropriate, given the explorative nature of this study. In this study we plan to use factor analysis to aggregate food consumption variables in order to derive a smaller set of 'dietary pattern' variables that capture as much of the variation in food intake as possible [10,36]. Our desire is to characterize the dietary behavior of the different ethnic groups; data driven approaches do not require a-prior theory of what patterns are expected.

As dietary pattern analysis has moved beyond North America and Europe, additional patterns have been described for ethnic or country-specific traditional diet. For example, a bean pattern - with high loading for legumes, tofu, and soy protein - was identified in a multiethnic cohort of Hawaiian women [37], while a traditional Korean pattern - with high loadings for soybean paste, anchovies, kimchi, and seaweed, and inverse an inverse loading for bread - was identified among Korean Americans in Michigan [38]. Traditional rice and bean patterns have been noted in several Latin American populations, including Brazilians [39] and in Puerto Ricans [40]. Ethnic or country-specific patterns demonstrate the diversity of dietary patterns that are followed, both within and between populations, and offer challenges for the assessment and interpretation of diet and health risk.

This study also provides the possibility to apply hypothesis-driven approaches which will be of interest when looking into the health consequences of the diet. When examining the dietary patterns of the Moroccan and Turkish individuals for example, the effect of the Mediterranean diet on health is of special interest. Although there is not one Mediterranean diet, it is described as being typically high in dietary fibre and n-3 fatty acids, as well as the consumption of fruit and vegetables and low consumption of saturated fat [41]. It has been found that a closer adherence to a Mediterranean style diet is linked to lower all-cause and cardiovascular mortality [42]. It will be interesting to study the differences in Mediterranean diet adherence between these two migrant groups and whether there is a beneficial link with cardiovascular health.

### Phase 4. Dietary patterns and health consequences

The use of dietary patterns can help to define dietary 'phenotypes' that are associated with high CVD risk among these groups. When looking into dietary pattern analysis, typically prudent patterns (or "healthier dietary patterns", high in vegetables, fruits, legumes, fish and whole grains) and western patterns (high in red meat, processed meat, butter, potatoes and high-fat dairy) have been found [10,43], although the specific foods contributing to each factor vary in level of contribution [11,36]. For example, Fung et al [43] reported that the prudent dietary pattern is correlated with a more favourable CVD risk profile and the Western dietary pattern diet with a less favourable CVD risk profile. This supports the use of such patterns to evaluate disease risk and, potentially, to serve as guidelines for healthy food consumption.

In phase 4, we specifically aim to address the relation between the ethnic-specific dietary patterns and risk factors for CVD. Data on risk factors for CVD will already be collected by the HELIUS study: trained staff will perform a physical examination in which data on body mass index (BMI), weight, abdominal and hip circumference, blood pressure (systolic and diastolic) will be collected. Additionally, fasting blood will be drawn to collect blood parameters relating to risk factors for CVD. Fasting glucose, HbA1c and lipid profile (concentrations of triglycerides, and total, LDL-, and HDL cholesterol) will be determined. Multiple linear regression analysis will allow the derived dietary patterns to be related to CVD risk factors within each ethnic group. The main outcomes will be blood pressure, blood glucose, blood lipid levels, body mass index, and waist and hip circumference. Potential confounders that will be considered including, lifestyle factors (smoking, physical activity), sex, age, as well as other explanatory variables such as SES, migration history and acculturation.

### Phase 5. Assessment of determinants of dietary patterns

Dietary habits are strongly influenced by ethnicity, culture and place of birth, and studies among non-western migrants in the Netherlands indicate that their diets differ from that of the ethnic Dutch population [15,16,44]. For example, Surinamese migrants seem to have more favourable fat intakes and consume more fruit, vegetables and fish [16]. However, despite more favourable intakes, the available data also indicate that migrants, like the host population, do not always meet guidelines for healthy eating, e.g. fruit and vegetable intakes are too low, and in some groups saturated fat intake is too high [15,16,44].

The process of migration implies a shift in environment which is likely to influence dietary intake; migration to a Western country may lead to adoption of the "Western" dietary pattern at the cost of the traditional diet, which had a presumably more favorable macronutrient composition. A recent review pointed out that following migration, the majority of ethnic groups alter their eating habits, combining parts of their traditional diet with some of the less healthy elements of the western diet [5]. Among Pakistani in Sweden for example, healthy foods such as vegetables, yoghurt, and fruit are being replaced by fruit syrups and ice cream, indicating a reduction in fiber and vitamin C and an increase in protein, saturated fat and sucrose [45].

The HELIUS-Dietary Patterns study will provide an opportunity to explore differences in dietary patterns between groups on the basis of their migration history (birth place, and length of residence) as well as their level of acculturation to Dutch society.

#### Dietary acculturation

Acculturation is complex and there are several theories regarding this process [46-51]. Regardless of which theory is used to describe acculturation, it is believed to occur at both the micro (individual) and macro (social/group) level. Acculturation at the individual level is referred to as "psychological acculturation" and refers to changes in attitudes, beliefs, behaviours, and values in individuals due to acculturation [49]. At the macro level, acculturation results in physical, biological, political, economical, and cultural changes in the acculturating group or in the society as a whole [49].

While acculturation pertains to adopting cultural traits, "dietary acculturation" specifically refers to the process that occurs when members of a migrating group adopt the eating patterns or food choices of their new environment [52]. Although results may vary due to the fact that the process of acculturation is very complex, in general, acculturation in non-western migrants in western countries has been linked with worse dietary choices including low consumption of fruits and vegetables, and higher intakes of fats and sweetened beverages [53]. In the same way, acculturation has been associated with lower physical activity, higher BMI and the likelihood of type-2 diabetes [4,54-57].

**Figure 1:**
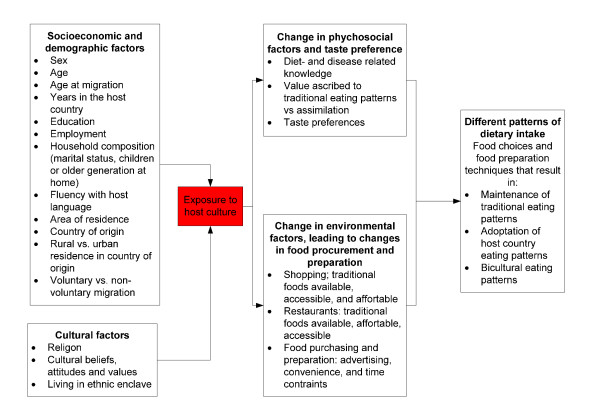
**Proposed model of dietary acculturation**. Footnote Figure 1: adapted from Satia-Abouta et al. [50]

Figure 1 shows a proposed model of dietary acculturation by Satia-Abouta et al [50]. This model hypothesizes that there is a complex and dynamic relationship of socioeconomic, demographic, and cultural factors with exposure to the host culture. According to Satia-Abouta, this set of characteristics predicts the extent to which new immigrants may change their attitudes and beliefs about food, taste preference, and food purchase and preparation. Ultimately, these factors can lead to changes in dietary intake [50]. Based on this model, we will collect data on socio-demographic, cultural factors, psychosocial factors, taste preference and environmental factors. Psychosocial factors will be measured by items about (1) diet- and disease related knowledge, attitudes, and beliefs, (2) value ascribed to traditional eating patterns versus assimilation [58] and (3) taste preference [59]. Environmental factors will be measured by items about (1) shopping; traditional foods available, accessible, and affordable, (2) restaurants: traditional foods available, assessable and affordable and (3) food purchasing and preparation.

**Figure 2:**
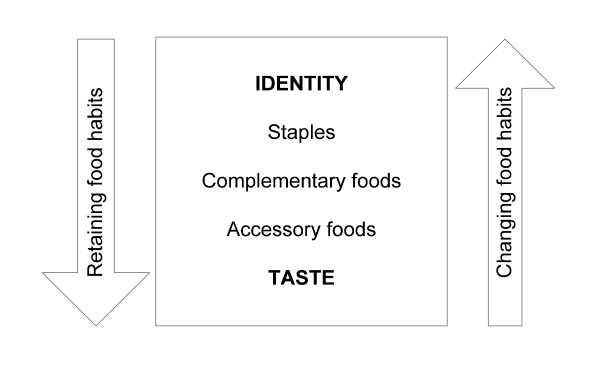
**The process of adaptation to a new dietary pattern after migration**. Footnote Figure 1: adapted from Koctürk-Runefors [47]

Phase 5 provides a unique opportunity to assess determinants of dietary patterns according to the model of Satia-Abouta and will allow research on how these determinants influence the maintenance of traditional eating patterns, the adoption of the Dutch/Western diet, or bicultural eating patterns. In addition, we plan to use the framework of Satia-Abouta to address how this change is characterized. This will be done by integrating the proposed model of adaptation by Koctürk (Figure 2). This model assumes that food habits change along an axis where identity and taste form the two extreme poles. When new foods are incorporated into the diet, the taste aspect is given priority, usually resulting in the adoption of accessory foods, which are tasty and often not considered as 'real food'. On the other hand the substitution of complementary foods takes place over a much longer period of time because the cultural identification with these foods is stronger, and the staples may remain the same for generations [47].

#### Sociodemographic characteristics and lifestyle

Data from studies in the Netherlands [14] and internationally [60] have suggested that lower SES groups are less likely to consume a healthy diet; dietary patterns of individuals with a higher SES are generally more in concordance with the guidelines for healthy eating as than those of lower SES [61]. However, Desilets et al reported a significant difference in SES score (built from education level, family income bracket, employment status at the time of the interview and housing status) across diet types of Haitians in Montreal, Canada. Subjects with a lower SES score, tended to score higher on the traditional cluster for which dietary quality was higher than the western type of diet [62]. This example points out that among migrants, looking into the determinants which predispose a certain dietary pattern is of crucial importance.

Among the ethnic Dutch population, a higher cosmopolitan-pattern score (greater intakes of fried vegetables, salad, rice, chicken, fish, and wine) was found to be associated with a higher educational level, more leisure physical activity, light cigarette smoking, and nutritional supplemental use [63]. A study among Surinamese migrants found that higher education level was not necessarily associated with a healthier diet [64], however, the measure of diet in this last study was limited. HELIUS-dietary patterns will allow more extensive study of the association of dietary patterns with sociodemographic and lifestyle factors in migrant origin populations in the Netherlands.

## Discussion

This study will provide important insight into the contribution of dietary patterns in the ethnic differences in disease risk. While there have been some studies that have compared dietary patterns from different countries, to date no single study in Europe has investigated the diet of different migrant groups residing in one setting. The heterogeneous nature of the study population provides variance in food intake, which is a great advantage when studying the link between diet and disease. Although heterogeneity can be achieved by making comparisons between countries, interpretation of their findings may be complicated as a result of confounding by differences in the local environment (e.g. climate, pollution, and urban density as well as the political context governing food regulations, health promotion). Studying a range of different ethnic groups in one setting limits the influence of these potential confounders on the relation between dietary patterns and risk factors for CVD. As such, this study will contribute to the understanding of how dietary choices influence the health of migrants compared to the ethnic Dutch population.

People choose foods and combinations of foods rather than isolated nutrients, and practical dietary advice to the public in terms of foods is preferred. Especially because dietary changes may be more readily achieved if recommended foods are compatible with existing patterns of food consumption. The information gathered will enable the setting of future priorities for public health nutrition.

## Abbreviations

CVD: Cardiovascular Disease; HELIUS: HEalthy LIfe in an Urban Setting; FFQ: Food Frequency Questionnaire; HDL: high density lipoprotein; BMI: Body Mass Index; SES: Socioeconomic Status

## Competing interests

The authors declare that they have no competing interests.

## Authors' contributions

LHD drafted the manuscript, conducts the study and contributed to the design. MN, JV, EJB, RMD, MBS, KS designed the study and they are members of the supervising board. MN, MBS, KS revised several early drafts of the paper and JV, EJB, MHB, HAMB, RMD commented on the final draft. All authors read and approved the final manuscript.

## Pre-publication history

The pre-publication history for this paper can be accessed here:

http://www.biomedcentral.com/1471-2458/11/441/prepub
